# Essential Oils from Four Brazilian *Piper* Species: GC-MS and HPTLC Profiling, Cytotoxicity Screening, and Behavioral Deterrence Against Imported Fire Ants

**DOI:** 10.3390/ph19091395

**Published:** 2026-09-03

**Authors:** Luciane Mendes Monteiro, Manuel Minteguiaga, Vera Lucia Pereira dos Santos, Sara Emilia Lima Tolouei, Shabana I. Khan, Mei Wang, Joseph Lee, Wilmer H. Perera, Paulo Vitor Farago, Farhan Mahmood Shah, Abbas Ali, Daniele Monteiro, Ikhlas Ahmed Khan, Sílvia Beatriz Bürger Tinelli, Arquimedes Gasparotto Junior, Jane Manfron

**Affiliations:** 1 Post Graduate Program in Pharmaceutical Sciences, State University of Ponta Grossa, Ponta Grossa 84030-900, Brazil; lmmonteiro@uepg.br (L.M.M.);; 2 Laboratory of Cardiovascular Pharmacology, Federal University of Grande Dourados, Dourados 79825-070, Brazil; 3 Espacio de Ciencia y Tecnología Química, Centro Universitario Regional Noreste, Universidad de la República, Tacuarembo 45000, Uruguay; manuel.minteguiaga@pedeciba.edu.uy; 4 Higher Education in Education, Humanities and Languages, Geosciences Area, Uninter International University Center, Curitiba 79801-015, Brazil; vera.sa@uninter.com; 5 Pharmacology Department, Federal University of Parana, Curitiba 81531-980, Brazil; saratolouei@gmail.com; 6 National Center for Natural Products Research, University of Mississippi, Oxford, MS 38655, USA; skhan@olemiss.edu (S.I.K.); farhanshah0009@yahoo.com (F.M.S.);; 7 United States Department of Agriculture, Agricultural Research Service (USDA-ARS), Natural Products Utilization Research Unit, University, MS 38677, USA; meiwang@olemiss.edu; 8 Camag Scientific, Inc., Wilmington, NC 28401, USA; 9 Rio de Janeiro Botanical Garden, Rio de Janeiro 22470-180, Brazil; 10 Post Graduation Program in Biotechnology Applied to Agriculture, Paranaense University, Umuarama 87502-210, Brazil; silvia.burger@edu.unipar.br

**Keywords:** behavioral deterrence, cytotoxicity, essential oils, Piperaceae, radical scavenging screening, *Solenopsis invicta*

## Abstract

**Background**: Brazilian *Piper* species are chemically diverse sources of volatile metabolites, yet direct comparative datasets integrating complementary analytical and biological endpoints remain limited. **Methods**: This study compared hydrodistilled essential oils (EOs) from *Piper amalago*, *P. diospyrifolium*, *P. grazielae*, and *P. solmsianum* using GC-MS, HPTLC fingerprinting, qualitative HPTLC-DPPH visualization, in vitro cytotoxicity screening, and behavioral assays with imported fire ants. **Results**: GC-MS revealed distinct chemical profiles among the four analyzed EOs. *P. solmsianum* was dominated by monoterpenes, mainly *β*-pinene (28.21%) and myrcene (24.73%); *P. diospyrifolium* was characterized by *trans*-*β*-caryophyllene (22.06%); and *P. grazielae* by germacrene D (14.06%), *trans*-*β*-caryophyllene (12.53%), and bicyclogermacrene (9.50%). *P. amalago* showed a mixed terpene profile containing bicyclogermacrene (11.00%), spathulenol (9.21%), and *β*-phellandrene (8.12%). HPTLC generated visually discriminative fingerprints that complemented the GC-MS profiles and identified chromatographic zones for subsequent compound-level characterization. Qualitative HPTLC-DPPH visualization revealed DPPH-reactive zones, most prominently in *P. grazielae*. The EOs produced moderate, cell-line-dependent cytotoxic responses, with *P. grazielae* showing the lowest IC_50_ values against BT-549 (45 µg/mL) and SK-OV-3 (43 µg/mL) cells and modest apparent selectivity indices (1.56–1.72). *P. amalago* reduced sand removal by hybrid imported fire ants at 31.25 µg/g, whereas *P. solmsianum* reduced sand removal by hybrid and red imported fire ants at 62.5 and 31.25 µg/g, respectively; neither oil caused mortality at 125 µg/g. **Conclusions**: The four EOs showed distinct chemical and biological profiles under the standardized conditions employed. *P. grazielae* showed the lowest IC_50_ values in the cytotoxicity screening and the most prominent DPPH-reactive chromatographic response, whereas *P. amalago* and *P. solmsianum* produced measurable nonlethal deterrence of fire ant digging behavior.

## 1. Introduction

The genus *Piper* L. (Piperaceae) is widely distributed throughout tropical and subtropical regions and represents the largest genus in the family, with particularly high species richness in South America and Brazil [1,2,3,4]. *Piper* species produce a broad range of specialized metabolites, including amides, alkaloids, lignans, phenylpropanoids, and terpenoids. These compounds contribute to ecological functions such as plant defense and have been investigated in association with antimicrobial, antioxidant, anti-inflammatory, cardiovascular, cytotoxic, and insect-related activities [2,5,6,7,8,9]. As emphasized by Salehi et al. [2], the biological significance of *Piper*-derived products is most appropriately interpreted in relation to the compound class, extract type, experimental model, and level of validation.

Among the natural products obtained from *Piper* species, essential oils (EOs) are particularly relevant because their volatile fractions are frequently rich in monoterpenes and sesquiterpenes. In plant–insect interactions, these constituents may influence orientation, host recognition, feeding, oviposition, nest-related behavior, and survival [6,10,11,12,13,14]. The chemical diversity and volatility of these mixtures make *Piper* EOs valuable systems for examining species-dependent patterns of insect response. Because EO activity may reflect the combined contribution of major and minor constituents within a specific biological context, integrating chemical profiling with standardized bioassays can identify differentiated response patterns and generate testable hypotheses for subsequent bioassay-guided and compound-level investigations.

Red imported fire ants (RIFA; *Solenopsis invicta* Buren) and hybrid imported fire ants (HIFA) are highly invasive pests of substantial ecological, agricultural, veterinary, and public health importance [10,15,16,17]. Their management relies extensively on synthetic insecticides and baits, while concerns regarding environmental exposure, effects on non-target organisms, and operational constraints have encouraged the investigation of botanical alternatives and behavior-oriented management strategies [11,12,13,14,17,18]. Behavioral endpoints are particularly informative because EOs may induce spatial avoidance or alter substrate-removal and digging behavior without causing acute mortality. Although related, repellency, digging deterrence, and acute toxicity should be treated as operationally distinct endpoints: repellency reflects avoidance of a treated substrate, digging deterrence reflects the suppression of substrate-removal activity, and acute toxicity reflects lethal effects. Their separate evaluation allows a more precise characterization of EO effects on imported fire ants and distinguishes nonlethal behavioral modulation from acute toxicity under controlled experimental conditions [8,14,17,18].

Previous studies have individually described the chemistry and selected bioactivities of *Piper amalago* L., *P. diospyrifolium* Kunth, *P. grazielae* Carv.-Silva & E.F.Guim. [synonym: *P. corcovadense* (Miq.) C.DC.], and *P. solmsianum* C.DC. (Supplementary Figure S1). These investigations have often involved plant material collected from different Brazilian regions and evaluated using different analytical or biological protocols. The present study provides a direct comparative evaluation of these four Brazilian *Piper* EOs within a single experimental framework that integrates GC-MS, HPTLC fingerprinting, HPTLC-DPPH visualization, cytotoxicity screening, and behavioral assays with imported fire ants. This standardized design enables the EOs to be compared under consistent analytical and biological conditions and reduces the methodological heterogeneity inherent in comparisons assembled from independent studies.

Gas chromatography–mass spectrometry (GC-MS) and high-performance thin-layer chromatography (HPTLC) provide complementary information for EO characterization. GC-MS supports the identification of volatile constituents through chromatographic behavior (linear retention indices) and mass-spectral data, which is complemented with the injection of authentic standards to confirm the proposed EOs compounds’ identity. HPTLC provides rapid, accessible, and visually discriminative fingerprints for direct sample comparison and visualization of chromatographic zones after derivatization. HPTLC-DPPH further enables qualitative localization of DPPH-reactive zones on the chromatographic plate, which preliminarily identify antiradical/antioxidant properties of EOs. Together with cytotoxicity and imported fire ant assays, these analytical approaches provide an integrated outline for recognizing chemical and biological patterns and prioritizing subsequent validation.

Accordingly, this study aimed to compare the volatile chemical profiles and biological screening responses of hydrodistilled EOs from *P. amalago*, *P. diospyrifolium*, *P. grazielae*, and *P. solmsianum*, four species occurring in the Atlantic Forest biome of Brazil. The integrated approach comprised GC-MS analysis, HPTLC fingerprinting, qualitative HPTLC-DPPH visualization, cytotoxicity screening, and behavioral assays with imported fire ants. We hypothesized that species-dependent differences in EO composition would be reflected in distinguishable chromatographic fingerprints and differentiated behavioral and cytotoxic responses. Evaluation within a single experimental design provides a standardized comparative dataset for chemical differentiation, biological prioritization, and the development of testable hypotheses for bioassay-guided and formulation-oriented investigations.

## 2. Results and Discussion

### 2.1. Yield and Features of EOs

Essential oils extracted from *P. amalago*, *P. diospyrifolium*, *P. grazielae*, and *P. solmsianum* exhibited a slightly yellowish coloration and a strong characteristic odor, in agreement with previous reports describing *Piper* EOs as pale yellow to yellowish and distinctly aromatic [19,20,21,22]. Yields varied among species, ranging from 0.50% (*v*/*w*) in *P. grazielae* to 1.12% (*v*/*w*) in *P. amalago*, with intermediate values for *P. solmsianum* (0.70%, *v*/*w*) and *P. diospyrifolium* (0.95%, *v*/*w*). Because drying and extraction (hydrodistillation) conditions were standardized, the observed differences are consistent with the influence of interspecific, environmental, and phenological factors previously reported for *Piper* EOs [5,23,24].

### 2.2. Chemical Profile of Piper EOs by GC-MS

Table 1 summarizes compounds present at ≥5% in at least one EO, whereas the complete compositional dataset is presented in Supplementary Table S1. *Piper solmsianum* showed the most evident divergence from the other EOs, with a monoterpene-rich profile dominated by *β*-pinene (28.21%) and myrcene (24.73%), followed by linalool (8.53%) and *α*-pinene (6.26%). In contrast, *P. diospyrifolium* was characterized by *trans*-*β*-caryophyllene (22.06%), *α*-pinene (7.55%), and α-copaene (7.10%). *Piper grazielae* was mainly sesquiterpene-rich, with germacrene D (14.06%), *trans*-*β*-caryophyllene (12.53%), and bicyclogermacrene (9.50%). *Piper amalago* showed a mixed terpene composition, with bicyclogermacrene (11.00%), spathulenol (9.21%), *β*-phellandrene (8.12%), and *α*-pinene (6.17%).

Within the standardized conditions of the present study, these profiles provide distinctly differentiated chemical signatures and a consistent comparative reference for the four analyzed samples.

Exploratory principal component analysis (PCA) and hierarchical clustering (Ward’s minimum variance method, Euclidean distance) were performed on the relative peak areas of major compounds (≥5% in at least one species) to visualize chemical similarity among the four *Piper* EOs. PCA (Supplementary Figure S2) separated *P. solmsianum* from the other three samples along PC1 (72.4% of total variance), associated with high relative abundances of *β*-pinene, myrcene, and linalool. PC2 (20.1% of variance) differentiated *P. amalago*, with positive loadings for bicyclogermacrene, spathulenol, and *β*-phellandrene, from *P. diospyrifolium*, with negative loadings for *α*-copaene and *trans*-*β*-caryophyllene; *P. grazielae* occupied an intermediate position. Hierarchical clustering (Supplementary Figure S3) reproduced this sample-level pattern: *P. diospyrifolium* and *P. grazielae* formed the closest pair, *P. amalago* joined this cluster at a greater distance, and *P. solmsianum* was the most chemically distinct sample.

The monoterpene-rich profile of *P. solmsianum* is consistent with reports from Southeastern Brazil describing monoterpene-predominant profiles for this species [25], whereas the sesquiterpene dominance of *P. diospyrifolium* and *P. grazielae* corroborates previous findings from Paraná State [26,27]. The mixed profile of *P. amalago* aligns with the chemical plasticity documented for this species across collection sites [23,24,28,29,30,31]. The present findings confirm and extend existing knowledge by providing a direct comparative dataset for four species analyzed under identical experimental conditions.

**Table 1:** Major constituents (≥5% in at least one sample) of essential oils from four Brazilian *Piper* species by GC-MS. The full compositional dataset is provided as Supplementary Table S1.

No.	Rt (min)	LRI ^a^	LRI ^b^	Compound	*P. amalago* (%)	*P. diospyrifolium* (%)	*P. grazielae* (%)	*P. solmsianum* (%)	Identification
2	18.351	930	932	*α*-Pinene *	6.17	7.55	3.47	6.26	tR, LRI, MS
5	20.908	969	974	*β*-Pinene *	3.77	0.55	3.59	28.21	tR, LRI, MS
6	21.780	982	988	Myrcene *	3.31	0.21	3.43	24.73	tR, LRI, MS
11	24.358	1020	1025	*β*-Phellandrene *	8.12	-	-	1.34	tR, LRI, MS
17	28.877	1083	1095	Linalool *	0.93	0.03	0.04	8.53	tR, LRI, MS
27	48.801	1374	1374	*α*-Copaene	0.31	7.10	1.39	0.74	LRI, MS
31	51.486	1416	1417	*trans*-*β*-Caryophyllene *	3.97	22.06	12.53	3.23	tR, LRI, MS
45	55.102	1472	1484	Germacrene D	0.78	3.19	14.06	-	LRI, MS
50	56.106	1488	1500	Bicyclogermacrene	11.00	1.27	9.50	1.64	LRI, MS
65	60.990	1557	1577	Spathulenol *	9.21	0.27	1.75	2.16	tR, LRI, MS

* Compound identification was confirmed with reference standards. Compound identification methods: tR, identification based on the retention time (tR) of genuine compounds on the DB-5MS column; MS, identification based on mass spectra search using Wiley and NIST libraries [32,33]; LRI, identification based on the comparison of calculated linear retention indices with the literature data. LRI ^a^: calculated retention index on DB-5MS column relative to n-alkanes. LRI ^b^, retention index reported in the literature [34].

### 2.3. High-Performance Thin-Layer Chromatography (HPTLC) Fingerprinting

**Figure 1:**
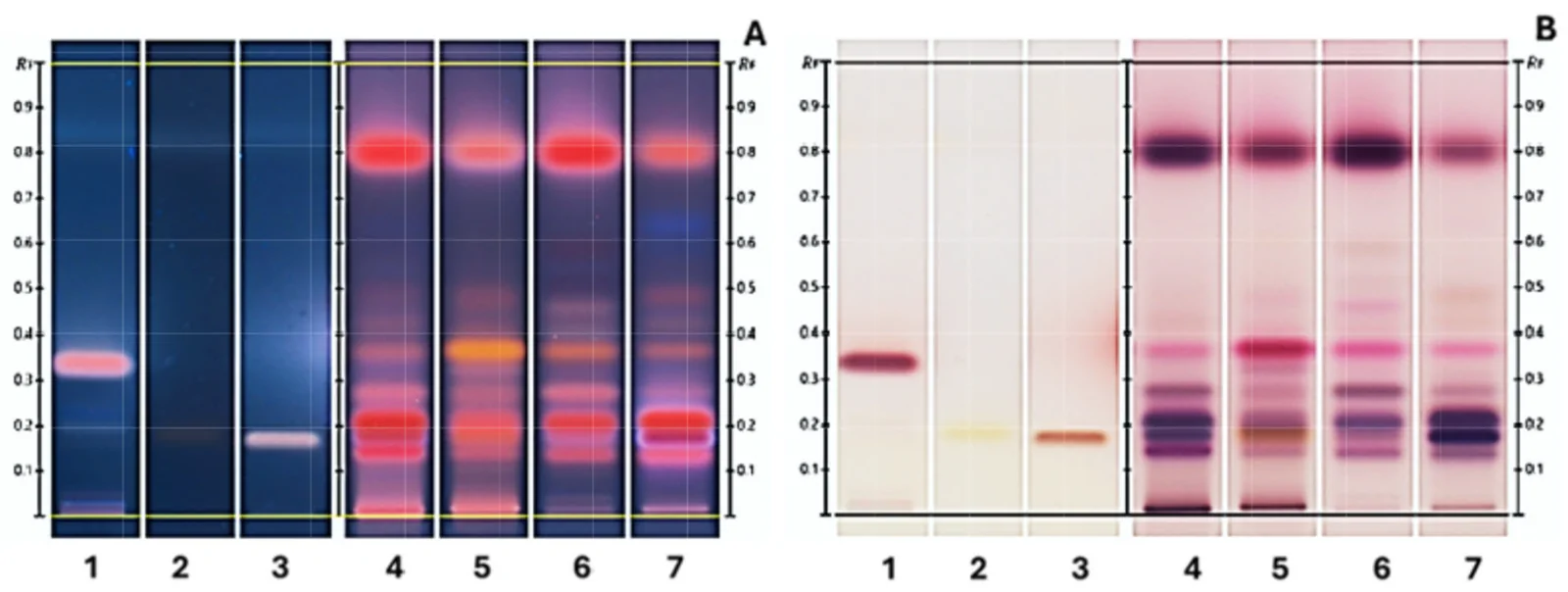
HPTLC fingerprints of essential oils from four *Piper* species under UV 366 nm (**A**) and white light remission transmission (**B**) post-derivatization with anisaldehyde–sulfuric acid reagent. Chromatographic system: ethyl acetate:toluene (5:95, *v*/*v*); EO solutions at 10 mg/mL applied to silica gel 60 F_254_ plates. Track 1: *α*-bisabolol, track 2: borneol, track 3: bisabolol oxide A, track 4: *Piper amalago*; track 5: *Piper diospyrifolium*; track 6: *Piper grazielae*; track 7: *Piper solmsianum*.

HPTLC fingerprinting provided an orthogonal and visually accessible comparison of the four EOs (Figure 1). After *para*-anisaldehyde–sulfuric acid derivatization, the oils showed distinguishable chromatographic patterns under UV 366 nm and white light, demonstrating the practical value of HPTLC as a rapid fingerprinting tool for comparative screening [35,36,37,38].

Three similar zones were found in the EOs of the four *Piper* EOs at *R*_F_ 0.14, *R*_F_ 0.37, and *R*_F_ 0.80 under UV 366 nm and white light after derivatization. The zone at *R*_F_ 0.37 was more intense in *P. diospyrifolium*, while the zone at *R*_F_ 0.80 was more intense in tracks corresponding to *P. amalago* and *P. grazielae*. Combining the two-detection mode post-derivatization, differences in the fingerprints of the four EOs are observed.

The retention-factor and post-derivatization color patterns indicated terpene-rich chromatographic zones, although individual assignments remain tentative. The visually differentiated zones complement the GC-MS profiles and provide practical targets for subsequent co-chromatography and compound-level characterization.

### 2.4. HPTLC-DPPH (2,2-diphenyl-1-picrylhydrazyl) Assay

**Figure 2:**
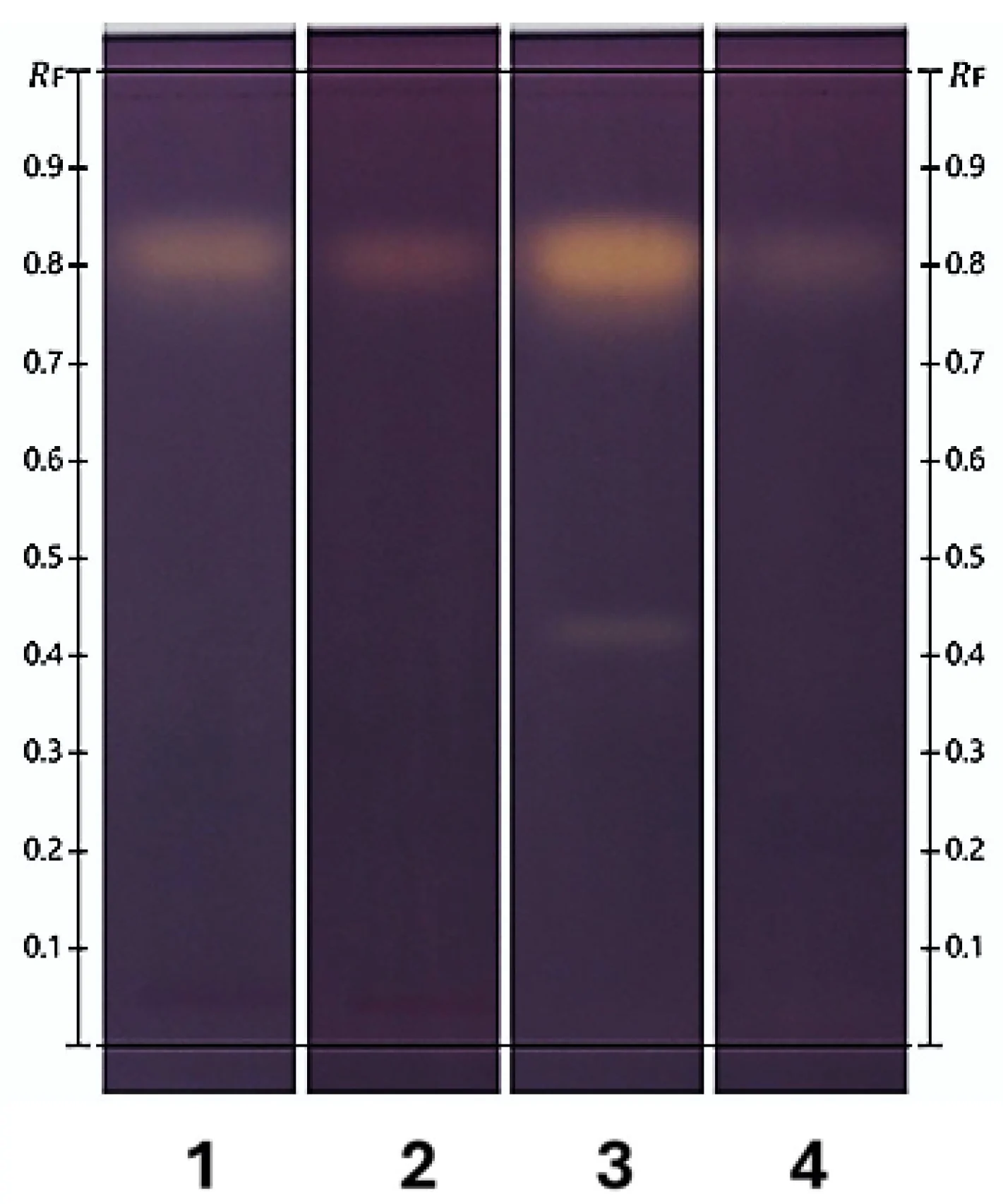
Qualitative HPTLC-DPPH radical-scavenging visualization of *Piper* essential oils under white light after derivatization with DPPH reagent. Yellowish zones indicate DPPH-reactive chromatographic regions, but the assay is not quantitative. Track 1: *Piper amalago*; track 2: *Piper diospyrifolium*; track 3: *Piper grazielae*; and track 4: *Piper solmsianum*.

All four EOs presented DPPH-reactive chromatographic zones, with *P. grazielae* showing the most intense and broadest zone on the plate (Figure 2). This qualitative visualization localizes regions with radical-scavenging reactivity and provides a practical basis for prioritizing chromatographic zones for subsequent chemical characterization.

### 2.5. Fire Ant Behavioral Deterrence and Acute Contact Toxicity

**Figure 3:**
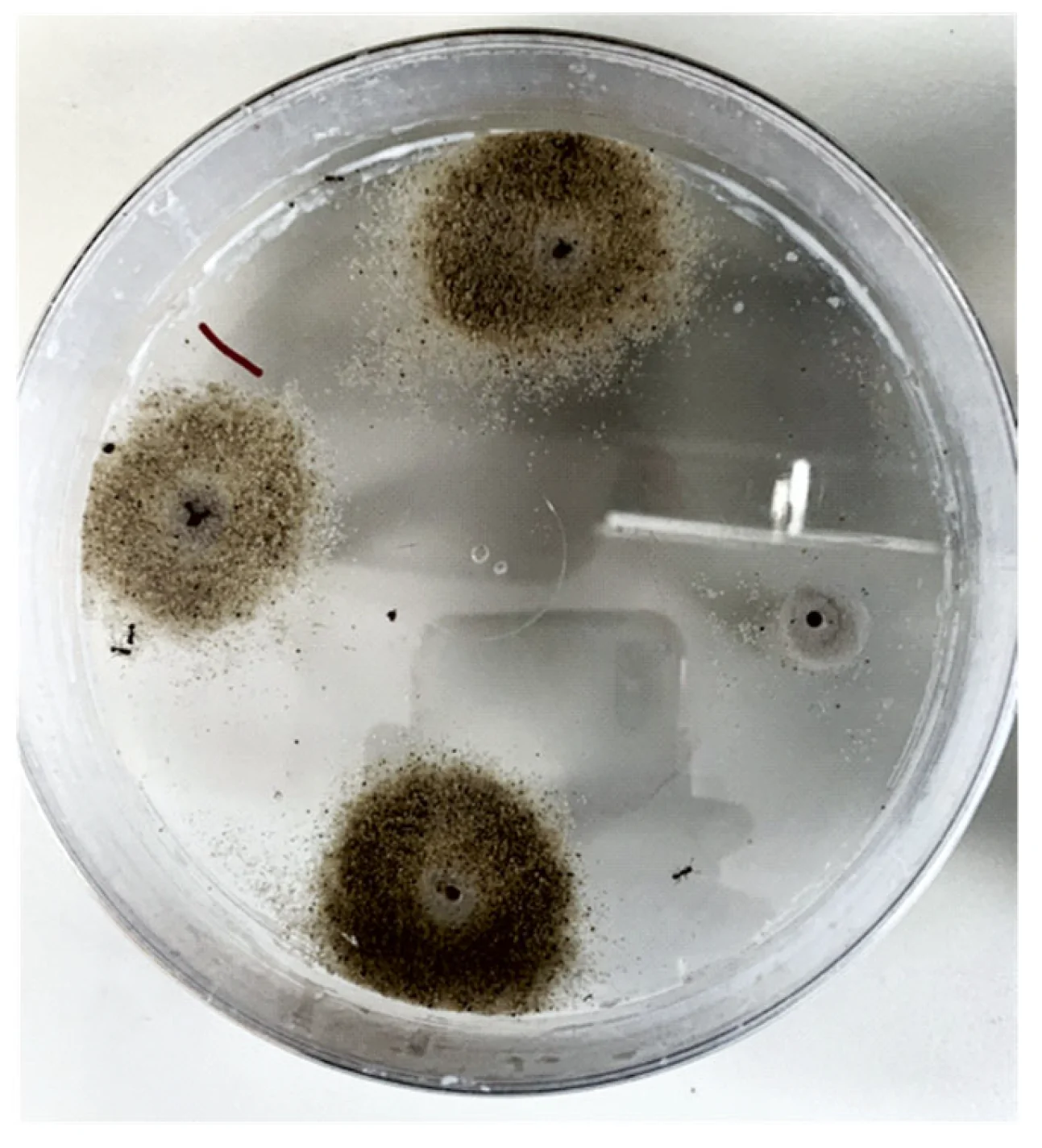
Experimental setup of the multiple-choice digging bioassay with imported fire ant workers. The arena contained four 2 mL vials filled with treated sand, with 50 workers released at the center. Digging deterrence was assessed by measuring sand removal after 24 h.

Initial screening indicated that *P. diospyrifolium* and *P. grazielae* did not produce sufficient behavioral responses to advance to concentration-response testing under the adopted workflow. *P. amalago* and *P. solmsianum* were therefore evaluated in multiple-choice digging assays with red imported fire ants (RIFA) and hybrid imported fire ants (HIFA) (Figure 3).

*Piper amalago* reduced sand removal by HIFA at concentrations of 31.25 µg/g and above. Against RIFA, reductions were detected at 62.5 and 125 µg/g, whereas 31.25 µg/g did not differ from the ethanol control. *Piper solmsianum* reduced sand removal by HIFA at 62.5 and 125 µg/g and by RIFA at 31.25 µg/g and above. DEET, used as a positive behavioral reference, reduced sand removal by HIFA at 31.25 µg/g and above and by RIFA at 62.5 and 125 µg/g (Table 2). These concentration-dependent effects demonstrate measurable digging suppression under the tested laboratory conditions.

No mortality was observed for either *P. amalago* or *P. solmsianum* EO at the highest concentration tested (125 µg/g). Together with the observed reductions in sand removal, this finding supports the interpretation that the effects reflected nonlethal behavioral modulation, without evidence of acute contact lethality under the conditions of the 24 h assay. For comparison, fipronil has previously shown LC_50_ values of 0.43 and 0.51 µg/g against RIFA and HIFA workers, respectively [8]. Reduced digging may reflect avoidance or deterrence; however, the underlying sensory or neurobehavioral mechanisms were not directly investigated.

The differentiated behavioral responses support subsequent bioassay-guided fractionation, isolated-constituent testing, and reconstructed-mixture experiments designed to identify the constituents and mixture interactions associated with digging suppression. Robust chemistry-behavior modeling will require a larger set of oils evaluated through comparable concentration-response assays.

### 2.6. Cytotoxicity Assay

The EOs produced moderate and cell-line-dependent cytotoxic responses (Table 3). *Piper grazielae* showed the lowest IC_50_ values against BT-549 (45 µg/mL) and SK-OV-3 (43 µg/mL), followed by *P. diospyrifolium* (62 and 60 µg/mL), *P. amalago* (71 and 73 µg/mL), and *P. solmsianum* (78 µg/mL for both cell lines). None of the EOs reached an IC_50_ against SK-MEL at concentrations up to 100 µg/mL, and only *P. grazielae* reached an IC_50_ against KB cells (86 µg/mL). Doxorubicin was used as the positive control, and the DMSO vehicle did not affect cell growth under the tested conditions.

Apparent selectivity indices were modest (Table 4). For *P. grazielae*, the indices ranged from 1.56 to 1.72, whereas the remaining oils generally showed values close to 1 or lower-bound estimates derived from censored LLC-PK1 IC_50_ values. These results prioritize *P. grazielae* for subsequent selectivity and compound-level evaluation while maintaining a screening-level interpretation of the observed cytotoxicity.

The differentiated chemical and cytotoxicity profiles nominate germacrene D, bicyclogermacrene, *α*-copaene, *trans*-*β*-caryophyllene, and spathulenol as candidates for isolated-compound and reconstructed-mixture testing, without assigning causality to any constituent. Follow-up studies should prioritize *P. grazielae* and evaluate selectivity in human non-tumor cells together with apoptosis, caspase, mitochondrial, cell-cycle, and clonogenic endpoints.

**Table 3:** Half-maximal inhibitory concentration (IC_50_, µg/mL) of *Piper* essential oils against tumor and non-tumor cell lines.

Samples	SK-MEL IC_50_ μg/mL	KB IC_50_ μg/mL	BT-549 IC_50_ μg/mL	SK-OV-3 IC_50_ μg/mL	LLC-PK1 IC_50_ μg/mL	Vero IC_50_ μg/mL
*P. amalago*	NC	NC	71	73	NC	70
*P. diospyrifolium*	NC	NC	62	60	NC	80
*P. grazielae*	NC	86	45	43	70	74
*P. solmsianum*	NC	NC	78	78	91	80

Notes: NC: IC_50_ > 100 µg/mL under the tested conditions (non-cytotoxic at the highest concentration evaluated). Tumor cell lines included SK-MEL (human melanoma), KB (human epidermoid carcinoma), BT-549 (human breast carcinoma), and SK-OV-3 (human ovarian adenocarcinoma). Non-tumor cell lines included LLC-PK1 (porcine kidney epithelial cells) and Vero (African green monkey kidney epithelial cells). Doxorubicin was used as the positive control. Experiments were performed in duplicate. IC_50_: half-maximal inhibitory concentration.

**Table 4:** Apparent selectivity indices calculated from available IC_50_ values.

Sample	SI LLC-PK1/BT-549	SI Vero/BT-549	SI LLC-PK1/SK-OV-3	SI Vero/SK-OV-3
*P. amalago*	>1.41	0.99	>1.37	0.96
*P. diospyrifolium*	>1.61	1.29	>1.67	1.33
*P. grazielae*	1.56	1.64	1.63	1.72
*P. solmsianum*	1.17	1.03	1.17	1.03

Notes: Values should be interpreted cautiously because several values are censored as NC (>100 µg/mL) and no confidence intervals were available. Abbreviations: BT-549: human breast cancer cells; LLC-PK1: porcine kidney epithelial cells; SK-OV-3: ovarian cancer cell line; Vero: renal epithelial cells; NC: IC_50_ > 100 µg/mL under the tested conditions; IC_50_: half-maximal inhibitory concentration.

### 2.7. Study Limitations

Several features define the scope of inference. Each species was represented by one population, one collection event, and one collection-derived extraction series, and only leaves were hydrodistilled, so the observed profiles should not be generalized as species-wide chemotypes. PCA and hierarchical clustering were exploratory because only four EO samples were analyzed. HPTLC band assignments were not confirmed by direct co-chromatography, isolation, densitometry, or HPTLC-MS, while HPTLC-DPPH provided qualitative localization rather than quantitative radical-scavenging potency. Cytotoxicity was evaluated as a duplicate screening assay without comprehensive mechanistic testing or an extensive panel of human non-tumor cells; several IC_50_ values were censored at >100 µg/mL, limiting apparent selectivity indices and precluding reliable confidence intervals. Detailed concentration-response digging data were available only for *P. amalago* and *P. solmsianum*, preventing robust compound-bioactivity correlation. Finally, the laboratory fire ant assays did not address formulation stability, persistence, environmental exposure, non-target effects, or field performance. These considerations delimit the scope of inference while preserving the internal comparative value of the standardized framework and define the next stages of compound-level, mechanistic, formulation, and field validation.

## 3. Materials and Methods

### 3.1. Chemicals

The insecticides DEET (CAS #: 134-62-3) and fipronil (CAS #: 120068-37-3) were purchased from Sigma-Aldrich (Saint Louis, MO, USA). The following reference terpene standards employed for identification of the components of *Piper* spp. EOs were supplied by Sigma-Aldrich: *α*-pinene (98%, CAS #: 80-56-8), camphene (>96%, CAS #: 79-92-5), sabinene (≥95.0%, CAS #: 3387-41-5), *β*-pinene (98.5%, CAS #: 127-91-3), myrcene (≥90.0%, CAS #: 123-35-3), *α*-phellandrene (≥85%, CAS #: 99-83-2), p-cymene (99%, CAS #: 99-87-6), 1,8-cineole (99%, CAS #: 470-82-6), limonene (>99%, CAS #: 138-86-3), *cis*- and *trans*-ocimene (mixture of isomers, ≥90%, CAS #: 13877-91-3), *γ*-terpinene (97%, CAS #: 99-85-4), terpinolene (≥95%, CAS #: 586-62-9), linalool (≥97%, CAS #: 78-70-6), *α*/*β*-thujone (~10% *β*-thujone, CAS # 76231-76-0), terpinen-4-ol (≥95%, CAS #: 562-74-3), *α*-terpineol (≥95.0%, CAS #: 98-55-5), bornyl acetate (95.0%, CAS #: 5655-61-8), *β*-elemene (>98%, CAS #: 33880-83-0), *α*-gurjunene (>97%, CAS #: 489-40-7), *trans-β*-caryophyllene (>80%, CAS #: 10579-93-8), valencene (≥80.0%, CAS #: 4630-07-3), elemicin (≥90.0%, CAS #: 487-11-6), caryophyllene oxide (95%, CAS #: 1139-30-6), globulol (≥98.5% sum of enantiomers, CAS #: 489-41-8), viridiflorol (95%, CAS #: 552-02-3), and *β*-eudesmol (≥90%, CAS #: 473-15-4). Analytical grade 3-carene (99.9%, CAS #: 13466-78-9) and *epi*-cedrol (99.9%, CAS #: 19903-73-2) were obtained from Agilent Technologies (Santa Clara, CA, USA), while *β*-phellandrene (≥95%, CAS #: 555-10-2), *α*-humulene (≥95%, CAS #: 6753-98-6), and *α*-bisabolol (≥95%, mixture of isomers, CAS #: 515-69-5) were obtained from Cayman Chemical (Ann Arbor, MI, USA) and borneol (≥98%, CAS #: 464-45-9) was obtained from Extrasynthese (Genay, France). Finally, *α*-thujene (>95%, CAS #: 2867-05-2), bisabolol oxide A (≥95%, 22567-36-8), and spathulenol (≥90%, CAS #: 6750-60-3) were supplied by BOC Science (New York, NY, USA).

Unless otherwise stated, the solvents employed in this study (methanol, ethanol, toluene, ethyl acetate, and n-hexane) were of HPLC grade and provided by Sigma-Aldrich. Sulfuric acid was also from Sigma-Aldrich, while glacial acetic acid was supplied by Supelco (Bellefonte, PA, USA).

### 3.2. Botanical Material

Samples of four *Piper* species were collected by Dr. Vera Lucia Pereira dos Santos and Dr. Jane Manfron: *P. amalago*, *P. diospyrifolium*, *P. grazielae*, and *P. solmsianum* (Supplementary Figure S1). Voucher specimens were prepared and deposited in the Herbarium of the Rio de Janeiro Botanical Garden, where their identities were confirmed by Dr. Daniele Monteiro. Collection data and voucher numbers are as follows: *Piper amalago*, Cerro Azul, Paraná, Brazil (24°18′ S, 49°37′ W; RB 903080); *P. diospyrifolium*, Paranaguá, Paraná, Brazil (25°28′34″ S, 48°41′07″ W; RB 903079); *P. grazielae*, Paranaguá, Paraná, Brazil (25°28′34″ S, 48°41′07″ W; RB 903078); and *P. solmsianum*, Ponta Grossa, Paraná, Brazil (25°05′23″ S, 50°06′23″ W; RB 903076).

### 3.3. Essential Oil (EO) Extraction by Hydrodistillation

The plant samples were oven-dried at 30 ± 5 °C. EOs were obtained from 100 g of fragmented dried leaves by hydrodistillation in a Clevenger-type apparatus for 4 h, in triplicate. After extraction, the EOs were collected, dried over anhydrous sodium sulfate (Na_2_SO_4_, Merck, Darmstadt, Germany), and stored at −4 ± 0.5 °C in the dark until analysis.

### 3.4. Chemical Profile of Essential Oils

#### 3.4.1. Gas Chromatography–Mass Spectrometry (GC-MS) Analysis

*Piper* EOs were analyzed by GC-MS using an Agilent 7890A GC system equipped with a 5975C quadrupole mass spectrometer and a 7693 autosampler (Agilent Technologies). Ten microliters of EOs were dissolved in 1 mL of *n*-hexane for each EO sample, and 1 µL of the sample solution was injected into the chromatographic system. The carrier gas used was helium (99.999%, nexAir, Memphis, TN, USA) with a flow rate of 1 mL/min, using a 25:1 split ratio, with the injector set at 260 °C. The separation of the volatile components of the EOs was achieved on a nonpolar DB-5MS column (Agilent J&W Scientific, Folsom, CA, USA), with dimensions of 60 m × 0.25 mm × 0.25 µm. The oven temperature program was as follows: 50 °C for 2 min, increased at 2 °C/min to 150 °C, then increased at 1 °C/min to 170 °C, and 8 °C/min to 250 °C. The total run time was 82 min. Each sample was injected in triplicate. Mass spectra were recorded at 70 eV electron ionization energy, in a scan mode from m/z 35 to 500. The ion source, quadrupole, and transfer line temperatures were maintained at 230, 150, and 280 °C, respectively.

Data acquisition was performed with the software Agilent MSD Chemstation (F.01.03.2357). The identification of the EO components involved comparing mass spectra with spectral databases (Wiley and the National Institute of Standards and Technology (NIST) [32,33], using a probability-based matching algorithm. Confirmation of the identification was based on linear retention indices (LRIs) compared to the literature [34], by injecting a solution of *n*-alkanes (C_8_–C_20_; Sigma-Aldrich) under the same analytical conditions previously described. The gross peak area percentage for each compound was obtained by full-scan GC/MS analysis on a DB-5MS column (analysis was performed at the National Center for Natural Products Research (NCNPR) at the University of Mississippi).

Three approaches for the identification of *Piper* EOs components were followed: #1. injection of authentic standards using the same analytical conditions as the samples; #2. comparison of mass spectra against databases (matching ≥90% of similarity) [32,33], probability-based match factor ≥ 90%; and #3. calculation of linear retention indices (LRIs) for each of the proposed components, and comparison with literature [34]. In the case of discrepancy between points #1 and #3, point #1 prevailed (always with the support of #2). This was the case for elemicin (a phenylpropanoid) and the oxygenated sesquiterpene *epi*-cedrol, where the differences between literature LRIs and experimental LRIs were 40 and 29 units (ΔLRIs in Table 1), respectively. Moreover, in general, the ΔLRIs calculated for the authentic standards were in the following ranges: 1 to 9 units for monoterpene hydrocarbons (values for *α*-thujene and *p*-cymene, respectively), 8 to 16 units for oxygenated monoterpenes (values for 1,8-cineole and *α*-terpineol/bornyl acetate, respectively), 1 to 12 units for sesquiterpene hydrocarbons (values for *trans*-*β*-caryophyllene and valencene, respectively), and finally, 17 to 24 units for oxygenated sesquiterpenes (values for viridiflorol and *α*-bisabolol, respectively). Thus, in the case of unavailable standards, the adopted identification criterion used point #2 (mass spectra comparison) and the upper limits of ΔLRIs for standards in the different terpene-subclasses (9 for monoterpene hydrocarbons, 16 for oxygenated monoterpenes, 12 for sesquiterpene hydrocarbons, and 24 for oxygenated sesquiterpenes). Despite efforts to identify most of the components of *Piper* spp. EOs, seven components remained unidentified (with abundances below 3%), and were labelled as such in Supplementary Table S1.

#### 3.4.2. High-Performance Thin-Layer Chromatography Fingerprinting

A quantity of 50 µL of the EOs was diluted with 950 µL of toluene and used for assessing the antiradical capacity of the EOs on the plate. For fingerprinting the EOs, samples were further diluted 2x with toluene. For standard preparation, 10 µL of *α*/*β*-thujone, bisabolol oxide A, and *α*-bisabolol (HWI Analytik, Rülzheim, Germany) were diluted in 990 µL of toluene. A stock solution of borneol (Extrasynthese) was prepared at 1 mg/mL in toluene and then diluted to 100 µg/mL.

An HPTLC system (CAMAG^®^, Wilmington, NC, USA) consisting of a TLC-Visualizer 2, an Automatic TLC Sampler 4 (ATS4), an Automatic Development Chamber 2 (ADC2), an Immersion Device III, and a TLC Plate Heater III, were used for the analysis. The visionCATS software version 3.1 was used to control the instruments and data processing. The parameters were chosen based on the USP general chapter <203> using Merck 20 × 10 cm silica gel F_254_ HPTLC plates (article No. 105642) as the stationary phase [39]. A quantity of 1 µL of the diluted EOs was applied onto the plate and developed with ethyl acetate and toluene 5:95 (*v*/*v*), then derivatized with *para*-anisaldehyde–sulfuric acid. A portion of 170 mL of methanol in a 250 mL glass flask was cooled, and then 20 mL of acetic acid, 10 mL of concentrated sulfuric acid, and 1 mL of *para*-anisaldehyde were carefully added and mixed to homogeneity. The plate was derivatized with an Immersion Device set at speed 5 and time 0 and heated at 100 °C for 3 min, then visualized at UV 366 nm and WLRT.

#### 3.4.3. HPTLC-DPPH

A quantity of 2 µL of the EOs was applied onto the plate, then developed with ethyl acetate and toluene 5:95 (*v*/*v*), then derivatized with a DPPH solution, as described by Andrade et al. [40], and visualized under WLRT after 30 min.

### 3.5. Fire Ant Behavioral Deterrence and Acute Contact Toxicity

#### 3.5.1. Ants

Red imported fire ants (RIFA; *Solenopsis invicta* Buren) and hybrid imported fire ants (HIFA; *S. invicta* Buren × *S. richteri* Forel) were used. HIFA workers were collected directly from natural mounds at the University Field Station (University of Mississippi, 15 County Road 2078, Abbeville, MS 38601, USA), whereas RIFA colonies were brought from Washington County, MS 38748, USA (33°09′31.2″ N 90°54′56.4″ W). The collections were contained in slippery-top plastic buckets and transferred to the laboratory, where they were maintained at 32 ± 2 °C temperature; 50 ± 10% relative humidity; and a 12:12 h (L:D) photoperiod. Crickets, a 25% sugar-water solution, and glass tubes filled halfway with water and plugged in with cotton were provided as a source of diet and moisture. Species and hybrids were identified on the basis of venom alkaloids and hydrocarbon indices of their workers [41].

#### 3.5.2. Digging Bioassay

*Piper amalago* and *P. solmsianum* EOs were evaluated using the multiple-choice digging bioassay described by Ali et al. [18]. The mass of sand removed from treated vials was used as an operational measure of digging deterrence. The repellency arena consisted of a 150 mm × 15 mm petri dish and four Nylgene Cryoware Cryogenic vials (2 mL each) with caps attached to the bottoms (Thermo Fisher Scientific, Cambridge, MA, USA). Four grams of sand (average 500 μm particle size) was treated in a volume of 400 µL of treatment solution in 45 mL fluted aluminum weighing dishes. All treatments were prepared in 100% ethanol. After evaporation of the solvent at room temperature, the sand was moistened with de-ionized water (0.6 µL/g) and packed in 2 mL vials, leaving no space. Fifty workers were released in the center of the arena and digging activity was evaluated at 24 h post-treatment. The remaining sand was collected back from treatment vials, oven-dried at 180 °C for 1 h, and weighed. DEET (*N*,*N*-diethyl-meta-toluamide) was employed as a positive control, while the sand treated with ethanol alone was the negative control. A series of serial dilutions were evaluated, starting from the highest concentration of 125 µg/g, until failure of the treatment. Three replications of the experiment were run on different days. The experimental conditions were set at 32 ± 2 °C temperature and 50 ± 10% relative humidity. Sand removal data were analyzed using one-way Analysis of Variance (ANOVA), and means were separated using the Ryan–Einot–Gabriel–Welsch multiple range test at *p* ≤ 0.05 (SAS 9.4 2012).

#### 3.5.3. Toxicity Bioassay

The toxicity of *P. amalago* and *P. solmsianum* EOs against RIFA and HIFA ants was investigated following Ali et al. [18]. Three grams of sand were placed in 45 mL fluted aluminum dishes and treated with 300 µL of the corresponding treatment solution. All treatments were prepared in 100% ethanol. After evaporation of the solvent, the sand was moistened with water (0.6 µL/g) and transferred into 60 × 15 mm stackable petri dish. Fipronil was used as a positive control, and sand treated with ethanol alone as negative control. Ten fire ant workers were released in treated sand placed in petri dishes. This was a contact bioassay in which mortality could result from exposure during digging or walking on the treated sand. A water-soaked cotton swab tip was placed in each petri dish to provide moisture. Dead ants were counted and recorded at 24 h post-treatment. Severely moribund ants were also treated as dead.

### 3.6. Cytotoxicity Assay

The cytotoxic potential of the EOs was evaluated against a panel of tumor and non-tumor cell lines. The tumor cell lines included SK-MEL (human melanoma, ATCC HTB-72), KB (human epidermoid carcinoma, ATCC CCL-17), SK-OV-3 (human ovarian adenocarcinoma, ATCC HTB-77), and BT-549 (human breast carcinoma, ATCC HTB-122). The non-tumor cell lines included LLC-PK1 (porcine kidney epithelial cells, ATCC CL-101) and Vero (African green monkey kidney epithelial cells, ATCC CCL-81). All cells were cultured in RPMI-1640 medium (pH 7.2) supplemented with 10% fetal bovine serum (FBS) and amikacin (60 µg/mL) and maintained at 37 °C in a humidified atmosphere containing 5% CO_2_. Cells were seeded into 96-well plates at a density of 1 × 10^4^ cells per well and incubated for 24 h to allow cell adhesion.

Stock solutions of the samples were prepared in dimethyl sulfoxide (DMSO) at 20 mg/mL and diluted in culture medium to obtain final concentrations of 100, 50, 25, 12.5, 6.25, and 3.13 µg/mL. The final DMSO concentration did not exceed 0.5% (*v*/*v*). The negative control consisted of culture medium containing the same final concentration of DMSO, whereas doxorubicin was used as the positive control. After 48 h of treatment, 10 µL of Cell Counting Kit-8 (CCK-8) reagent was added to each well, followed by an additional incubation for 45 min to 1 h. Absorbance was then measured at 450 nm using a microplate reader.

Cell viability was determined relative to the DMSO control. Half-maximal inhibitory concentration (IC_50_) values were calculated from dose–response curves using nonlinear regression analysis and expressed as µg/mL. When 50% inhibition was not reached at the highest concentration tested, the result was reported as IC_50_ > 100 µg/mL. The assay was conducted at the National Center for Natural Products Research, University of Mississippi, Oxford, MS, USA.

### 3.7. Statistical and Exploratory Data Analysis

GC-MS compositional data were summarized descriptively using relative peak areas. Exploratory principal component analysis (PCA) and hierarchical clustering were conducted using the relative peak areas of the ten compounds present at ≥5% in at least one EO sample (Table 1). Compounds not detected in a sample were entered as zero. Data were mean-centered and scaled to unit variance before PCA. Analyses were performed using R, version 4.2 (R Core Team, 2023), and the factoextra package, version 1.0.7, was used for visualization. Hierarchical clustering was performed using Euclidean distance and Ward’s method. Results are presented in Supplementary Figures S2 and S3. These analyses describe only the four EO samples examined, each representing one population and collection event, and do not support population-level generalization, taxonomic inference, or associations with the biological endpoints.

HPTLC fingerprints and HPTLC-DPPH results were evaluated qualitatively. No inferential statistical comparisons were performed for these analyses.

Digging-bioassay results were expressed as mean ± standard error (SE). Three experimental replications were conducted on different days. The response variable was the mass of sand removed from each treatment vial after 24 h. Separate results were reported for HIFA and RIFA, for each test material, and for the concentration sets designated Experiment 1 and Experiment 2. Differences among the ethanol control and the three concentrations within each analysis were evaluated by analysis of variance using SAS software, version 9.4. The Ryan–Einot–Gabriel–Welsch multiple range test was used to generate the mean-grouping letters reported in Table 2. Statistical significance was defined as *p* ≤ 0.05.

For the cytotoxicity assays, cell viability was calculated relative to the DMSO vehicle control. Half-maximal inhibitory concentrations (IC_50_) were estimated from concentration–response data using nonlinear regression. When 50% inhibition was not reached at the highest tested concentration, the result was reported as IC_50_ > 100 µg/mL. Apparent selectivity indices were calculated as the ratio of the IC_50_ for a non-tumor cell line to the IC_50_ for the corresponding tumor cell line. When the non-tumor IC_50_ was reported as >100 µg/mL and the tumor-cell IC_50_ was estimable, the resulting selectivity index was reported as a lower bound and retained the “>” symbol.

## 4. Conclusions

Under the standardized conditions used in this study, the four Brazilian *Piper* EOs exhibited distinct chemical and biological profiles. *Piper solmsianum* showed a monoterpene-rich profile dominated by *β*-pinene (28.21%) and myrcene (24.73%), whereas *P. diospyrifolium* and *P. grazielae* were characterized predominantly by sesquiterpenes, particularly *trans-β*-caryophyllene (22.06%) in *P. diospyrifolium* and germacrene D (14.06%) and *trans-β*-caryophyllene (12.53%) in *P. grazielae*. *Piper amalago* exhibited a mixed terpene profile, with bicyclogermacrene (11.00%), spathulenol (9.21%) and *β*-phellandrene (8.12%) among its major constituents. HPTLC provided visually distinguishable fingerprints that complemented the GC-MS profiles, while qualitative HPTLC-DPPH analysis revealed DPPH-reactive zones in all four EOs, with the most prominent response observed for *P. grazielae*.

In the cytotoxicity screening, *P. grazielae* showed the lowest IC_50_ values against BT-549 (45 µg/mL) and SK-OV-3 (43 µg/mL) cells and was the only EO to reach an IC_50_ against KB cells (86 µg/mL). However, its apparent selectivity indices were modest (1.56–1.72). None of the four EOs reached an IC_50_ against SK-MEL cells at concentrations up to 100 µg/mL. These findings therefore indicate differentiated, cell-line-dependent cytotoxic responses at the screening level rather than evidence of selective anticancer activity.

In the fire ant assays, *P. amalago* and *P. solmsianum* produced concentration-dependent reductions in sand removal. *Piper amalago* reduced digging by hybrid imported fire ants from 31.25 µg/g and by red imported fire ants at 62.5 and 125 µg/g, whereas *P. solmsianum* reduced digging by hybrid imported fire ants at 62.5 and 125 µg/g and by red imported fire ants from 31.25 µg/g. These effects occurred within the concentration range in which DEET, used as the positive behavioral reference, also reduced sand removal. Neither EO caused mortality at 125 µg/g during the 24 h assay, indicating that the observed responses were associated with nonlethal digging deterrence rather than acute contact toxicity under the tested conditions.

Overall, the results demonstrate species-dependent chemical fingerprints and differentiated biological responses among the four *Piper* EOs under a standardized comparative framework. *Piper grazielae* was distinguished by its lower IC_50_ values in the cytotoxicity screening and its more prominent DPPH-reactive chromatographic zones, whereas *P. amalago* and *P. solmsianum* showed measurable nonlethal effects on fire ant digging behavior. These findings identify specific EOs and biological endpoints for subsequent compound-level and mechanistic investigation, without assigning the observed activities to individual EO constituents.

## Figures and Tables

**Table 2:** Mean weight (g) of treated sand removed by imported fire ant workers released in a multiple-choice digging bioassay with different concentrations of *P. amalago* and *P. solmsianum* essential oils and DEET.

Concentration (µg/g)	Mean ± SE (HIFA)	F-Value	*p*-Value	Mean ± SE (RIFA)	F-Value	*p*-Value
*P. amalago* EO—Experiment 1						
Control	1.30 ± 0.06 a	3.023	0.071			
15.6	0.96 ± 0.17 a					
7.8	0.78 ± 0.15 a					
3.9	1.01 ± 0.07 a					
*P. amalago* EO—Experiment 2						
Control	2.06 ± 0.01 a	32.094	<0.001	1.87 ± 0.06 a	16.302	<0.001
125	0.27 ± 0.14 c			0 ± 0 c		
62.5	0.20 ± 0.19 c			0.67 ± 0.30 b		
31.25	1.01 ± 0.19 b			1.39 ± 0.26 ab		
*P. solmsianum* EO—Experiment 1						
Control				1.95 ± 0.14 a	2.564	0.128
15.6				1.33 ± 0.14 a		
7.8				1.56 ± 0.17 a		
3.9				1.48 ± 0.20 a		
*P. solmsianum* EO—Experiment 2						
Control	1.50 ± 0.21 a	10.956	0.003	1.94 ± 0.12 a	21.352	<0.001
125	0.27 ± 0.14 b			0 ± 0 c		
62.5	0.29 ± 0.29 b			0.65 ± 0.29 b		
31.25	1.25 ± 0.07 a			1.23 ± 0.18 b		
DEET—Experiment 1						
Control	1.26 ± 0.19 a	0.24	0.870	—	—	—
15.6	0.98 ± 0.49 a			—		
7.8	1.37 ± 0.28 a			—		
3.9	1.16 ± 0.29 a			—		
DEET—Experiment 2						
Control	1.58 ± 0.11 a	9.71	0.005	1.43 ± 0.19 a	16.24	0.001
125	0.42 ± 0.25 b			0.08 ± 0.04 c		
62.5	0.87 ± 0.13 b			0.74 ± 0.18 b		
31.25	0.84 ± 0.04 b			1.14 ± 0.10 ab		

Each individual experiment consisted of three concentrations and a control (*n* = 3 independent replicates). Experiments continued until a failure point was reached. — no experiments were conducted. Means within the same column and experiment followed by different letters differ significantly according to the Ryan–Einot–Gabriel–Welsch multiple range test (*p* ≤ 0.05).

## Data Availability

The data supporting the reported results are available within the article and its Supplementary Materials. Additional raw data are available from the corresponding author.

## Supplementary Materials

The following supporting information can be downloaded at: https://www.mdpi.com/article/10.3390/ph19091395/s1, Figure S1: Representative specimens of the four *Piper* species investigated in this study: (a) *Piper amalago* L.; (b) *Piper diospyrifolium* Kunth; (c) *Piper grazielae* Carv.-Silva & E.F.Guim.; and (d) *Piper solmsianum* C.DC. Scale bars = 10 cm. Photographs by Dr. Jane Manfron; Table S1: GC-MS composition and relative peak areas of essential oils from four Brazilian *Piper* species; Figure S2: Exploratory PCA biplot of the four essential-oil samples based on the relative peak areas of compounds present at ≥5% in at least one sample. The analysis provides a descriptive visualization of chemical similarity among the four samples and should not be interpreted as evidence of species-level chemotypes; Figure S3: Exploratory hierarchical clustering dendrogram of the four essential-oil samples based on the standardized relative peak areas of compounds present at ≥5% in at least one sample. Clustering was performed using Ward’s linkage and Euclidean distance. The dendrogram is intended as a descriptive representation of chemical similarity among the analyzed samples.
