# Supplementary material for: Essential Oils from Four Brazilian Piper Species: GC-MS and HPTLC Profiling, Cytotoxicity Screening, and Behavioral Deterrence Against Imported Fire Ants
Source: Pharmaceuticals (Basel). 2026 Sep 3;19(9):1395. doi: 10.3390/ph19091395 (PMC13610860; doi:10.3390/ph19091395)

Essential Oils from Four Brazilian *Piper* Species: GC-MS and HPTLC Profiling, Cytotoxicity Screening, and Behavioral Deterrence Against Imported Fire Ants

Luciane Mendes Monteiro [1,2], Manuel Minteguiaga [3], Vera Lucia Pereira dos Santos [4], Sara Emilia Lima Tolouei [5,6], Shabana I. Khan [6], Mei Wang [7], Joseph Lee [6], Wilmer H. Perera [8], Paulo Vitor Farago [1,6], Farhan Mahmood Shah [6], Abbas Ali [6], , Daniele Monteiro [9], Ikhlas Ahmed Khan [6], Sílvia Beatriz Bürger Tinelli [10], Arquimedes Gasparotto Junior [2,10,*] and Jane Manfron [1,6]

1 Post Graduate Program in Pharmaceutical Sciences, State University of Ponta Grossa, Ponta Grossa, 84030-900, Brazil; lmmonteiro@uepg.br (L.M.M.)
2 Laboratory of Cardiovascular Pharmacology, Federal University of Grande Dourados, Dourados, 79825-070,Brazil
3 Espacio de Ciencia y Tecnología Química, Centro Universitario Regional Noreste, Universidad de la República, Tacuarembo 45000, Uruguay; manuel.minteguiaga@pedeciba.edu.uy
4 Higher Education in Education, Humanities and Languages, Geosciences Area, Uninter International University Center, Curitiba, 79801-015, Brazil; vera.sa@uninter.com
5 Pharmacology Department, Federal University of Parana, Curitiba, Brazil; saratolouei@gmail.com
6 National Center for Natural Products Research, University of Mississippi, Oxford, MS 38655, USA; skhan@olemiss.edu (S.I.K.); farhanshah0009@yahoo.com (F.M.S.)
7 United States Department of Agriculture, Agricultural Research Service (USDA-ARS), Natural Products Utilization Research Unit, University, MS 38677, USA; meiwang@olemiss.edu (M.W.)
8 Camag Scientific, Inc, Wilmington, 28401, USA
9 Rio de Janeiro Botanical Garden, Rio de Janeiro, 22470-180, Brazil
10 Post Graduation Program in Biotechnology Applied to Agriculture, Paranaense University, Umuarama, 87502-210, Brazil; silvia.burger@edu.unipar.br
* Correspondence: arquimedesgasparotto@gmail.com; Tel.: +55-67-98115-9006

Supplementary Materials: Figure S1: Representative specimens of the investigated species; Table
S1: Complete GC-MS composition; Figure S2: PCA biplot; Figure S3: Hierarchical clustering dendrogram.

Figure S1. Representative specimens of the four *Piper* species investigated in this study: (a) *Piper amalago* L.; (b) *Piper diospyrifolium* Kunth; (c) *Piper grazielae* Carv.-Silva & E.F.Guim.; and (d) *Piper solmsianum* C.DC. Scale bars = 10 cm. Photographs by Dr. Jane Manfron.

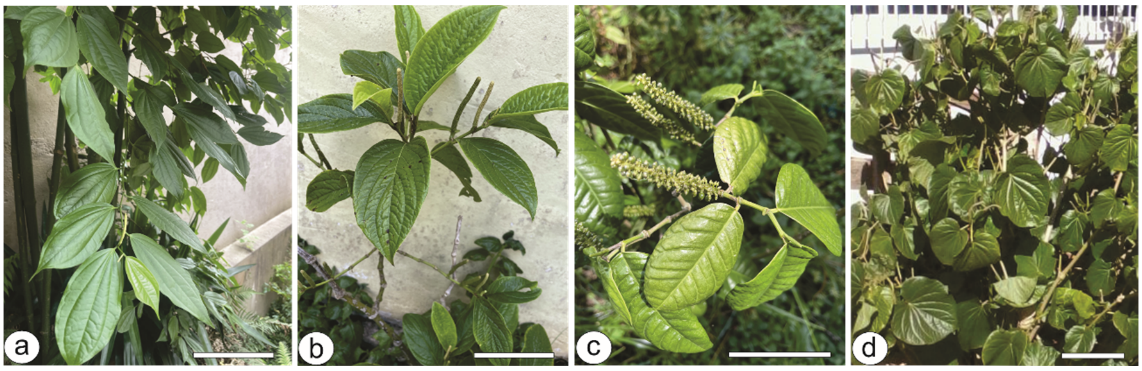

Table S1. GC-MS composition and relative peak areas of essential oils from four Brazilian *Piper* species.

No.	Rt (min)	LRI[a]	LRI[b]	ΔLRI	Compound Identification	P. amalago	P. diospyrifolium	P. grazielae	P. solmsianum	ID
						%	%	%	%	
1	17.870	923	924	1	α-Thujene*	0.06	-	0.05	0.36	tR, LRI, MS
2	18.351	930	932	2	α-Pinene*	**6.17**	**7.55**	3.47	**6.26**	tR, LRI, MS
3	19.137	942	946	4	Camphene*	1.15	0.16	0.19	0.27	tR, LRI, MS
4	20.635	965	969	4	Sabinene*	0.18	-	0.04	0.26	tR, LRI, MS
5	20.908	969	974	5	β-Pinene*	3.77	0.55	3.59	**28.21**	tR, LRI, MS
6	21.780	982	988	6	Myrcene*	3.31	0.21	3.43	**24.73**	tR, LRI, MS
7	22.700	996	1002	6	α-Phellandrene*	0.41	0.13	0.14	0.14	tR, LRI, MS
8	23.256	1004	1008	4	3-Carene*	0.11	0.24	0.04	0.03	tR, LRI, MS
9	23.737	1011	1020	9	p-Cymene*	0.98	0.38	0.15	1.12	tR, LRI, MS
10	24.283	1018	1026	8	1,8-Cineole*	-	0.22	0.51	-	tR, LRI, MS
11	24.358	1020	1025	5	β-Phellandrene*	**8.12**	-	-	1.34	tR, LRI, MS
12	24.438	1021	1024	3	Limonene*	1.58	3.52	0.79	1.40	tR, LRI, MS
13	24.855	1027	1032	5	cis-β-Ocimene*	0.01	0.60	0.09	0.03	tR, LRI, MS
14	25.631	1037	1044	7	trans-β-Ocimene*	0.03	0.16	0.21	0.02	tR, LRI, MS
15	26.395	1048	1054	6	γ-Terpinene*	0.02	0.03	0.02	0.30	tR, LRI, MS
16	28.503	1078	1086	8	Terpinolene*	0.01	0.03	0.02	0.14	tR, LRI, MS
17	28.877	1083	1095	12	Linalool*	0.93	0.03	0.04	**8.53**	tR, LRI, MS
18	31.894	1126	1140	14	trans-Verbenol	0.03	0.03	-	0.03	LRI, MS
19	34.279	1159	1174	15	Terpinen-4-ol*	0.24	0.04	0.02	1.30	tR, LRI, MS
20	35.023	1170	1186	16	α-Terpineol*	0.21	0.06	0.06	0.27	tR, LRI, MS
21	41.768	1268	1284	16	Bornyl acetate*	0.08	-	0.02	0.38	tR, LRI, MS
22	44.222	1308	1324	16	Myrtenyl acetate	-	0.30	0.01	0.03	LRI, MS
23	45.544	1324	1335	11	δ-Elemene	0.12	-	0.08	0.01	LRI, MS
24	46.175	1334	-	-	Unidentified 1 (Sesquiterpene hydrocarbon)	1.84	0.29	1.64	0.18	-
25	47.047	1347	1345	-2	α-Cubebene	0.13	0.14	0.55	0.07	LRI, MS
26	48.223	1365	1369	4	Cyclosativene	0.03	0.22	0.08	0.02	LRI, MS
27	48.801	1374	1374	0	α-Copaene	0.31	**7.10**	1.39	0.74	LRI, MS
28	49.256	1381	1387	6	β-Bourbonene	0.12	0.04	0.16	-	LRI, MS
29	49.571	1386	1389	3	β-Elemene*	0.88	2.26	2.27	0.02	tR, LRI, MS
30	50.946	1407	1409	2	α-Gurjunene*	0.10	0.14	0.34	0.12	tR, LRI, MS

31	51.486	1416	1417	1	*trans*-*β*-Caryophyllene*	3.97	**22.06**	**12.53**	3.23	*tR*, LRI, MS
32	51.957	1423	1430	7	*β*-Copaene	0.12	0.26	0.83	0.13	LRI, MS
33	52.096	1425	1434	9	ɤ-Elemene (Elixene)	-	1.77	0.30	0.01	LRI, MS
34	52.251	1428	1431	3	*β*-Gurjunene	0.02	-	0.14	0.02	LRI, MS
35	52.422	1430	1432	2	*trans*-*α*-Bergamotene	0.08	3.74	0.11	0.05	LRI, MS
36	52.636	1434	1439	5	Aromandendrene	0.75	1.05	0.37	1.15	LRI, MS
37	52.700	1435	1442	7	Guaia-6,9-diene	0.03	-	0.61	-	LRI, MS
38	53.032	1440	1445	5	*epi*-*β*-Santalene	0.03	0.09	-	0.14	LRI, MS
39	53.053	1442	1454	12	*α*-Patchoulene	-	-	-	0.14	LRI, MS
40	53.444	1446	1452	6	*α*-Humulene*	0.69	3.15	3.55	0.25	*tR*, LRI, MS
41	53.797	1452	1457	5	*β*-Santalene	0.43	0.78	-	-	LRI, MS
42	53.914	1453	1464	9	9-*epi*-*trans*-Caryophyllene	-	2.47	3.26	0.14	LRI, MS
43	54.615	1464	1475	11	*γ*-Gurjunene	-	0.07	0.24	0.04	LRI, MS
44	54.775	1467	1478	11	*γ*-Muurolene	2.05	1.35	2.56	0.08	LRI, MS
45	55.102	1472	1484	12	Germacrene D	0.78	3.19	**14.06**	-	LRI, MS
46	55.449	1477	1489	12	*β*-Selinene (*β*-Eudesmene)	0.05	0.71	0.58	0.08	LRI, MS
47	55.722	1481	1492	11	*δ*-Selinene	0.16	-	0.57	0.12	LRI, MS
48	55.845	1483	1493	10	*epi*-Cubebol	-	0.34	-	0.16	LRI, MS
49	55.872	1484	1496	12	Valencene*	0.87	-	1.79	-	*tR*, LRI, MS
50	56.106	1488	1500	12	Bicyclogermacrene	**11.00**	1.27	**9.50**	1.64	LRI, MS
51	56.305	1490	1500	10	*α*-Muurolene	0.85	1.60	2.15	0.35	LRI, MS
52	56.867	1499	1505	6	*β*-Bisabolene	2.90	0.66	0.35	0.03	LRI, MS
53	57.134	1503	1513	10	*γ*-Cadinene	1.47	0.44	1.33	0.24	LRI, MS
54	57.364	1509	1521	12	*trans*-Calamenene	0.33	0.22	2.34	0.10	LRI, MS
55	57.738	1511	1522	11	*δ*-Cadinene	4.49	2.77	3.39	1.06	LRI, MS
56	58.001	1515	1555	40	Elemicin*	0.01	-	0.22	-	*tR*, MS
57	58.359	1521	1533	12	*trans*-Cadina-1,4-diene (*trans*-Cubenene)	0.06	0.15	0.43	0.02	LRI, MS
58	58.498	1522	1539	17	*α*-Copaen-11-ol	-	0.09	0.43	-	LRI, MS
59	58.755	1526	1537	11	*α*-Cadinene	0.23	0.09	0.17	-	LRI, MS
60	59.001	1529	1548	19	Elemol	0.04	-	0.11	0.05	LRI, MS
61	58.605	1532	1544	12	*α*-Calacorene	-	0.48	0.19	-	LRI, MS
62	60.147	1547	1559	12	Germacrene B	3.47	1.75	3.31	0.57	LRI, MS
63	60.637	1552	1570	18	Caryophyllenyl alcohol	-	0.22	0.06	-	LRI, MS
64	60.798	1554	1567	13	Palustrol	0.35	0.21	0.16	0.07	LRI, MS
65	60.990	1557	1577	20	Spathulenol*	**9.21**	0.27	1.75	2.16	*tR*, LRI, MS
66	61.440	1563	1582	19	Caryophyllene oxide*	1.16	3.26	1.07	0.42	*tR*, LRI, MS
67	61.729	1568	1590	22	Globulol*	1.04	1.16	0.70	0.65	*tR*, LRI, MS

68	62.251	1575	1592	17	Viridiflorol*	0.42	1.31	0.35	0.08	*tR*, LRI, MS
69	62.435	1577	1595	18	Cubeban-11-ol	0.66	0.17	0.11	0.12	LRI, MS
70	62.905	1584	1600	16	Rosifoliol	0.16	-	0.12	0.07	LRI, MS
71	63.098	1587	1602	15	Ledol	0.47	0.99	0.76	0.41	LRI, MS
72	63.274	1589	1618	29	*epi*-Cedrol*	0.04	1.08	0.05	0.08	*tR*, MS
73	63.579	1594	1618	24	Junenol	-	0.29	0.08	0.01	LRI, MS
74	63.916	1598	1599	1	Widdrol	0.22	0.37	0.14	0.36	LRI, MS
75	64.149	1601	1622	21	10-*epi*-γ-Eudesmol	0.10	-	0.24	0.11	LRI, MS
76	64.547	1606	1630	24	Muurola-4,10(14)-dien-1β-ol	0.22	1.80	0.42	0.06	LRI, MS
77	64.832	1610	1618	8	1,10-di-*epi*-cubenol	0.29	1.80	0.42	0.18	LRI, MS
78	64.970	1612	1627	15	1-*epi*-cubenol	0.16	-	0.17	0.51	LRI, MS
79	65.211	1615	-	-	Unidentified 2 (Oxygenated sesquiterpene)	2.54	1.77	0.22	0.18	-
80	65.606	1620	1640	20	*epi*-α-Muurolol (τ-Muurolol)	3.14	0.87	0.85	0.37	LRI, MS
81	65.815	1623	1644	21	α-Muurolol (δ-Cadinol)	0.57	0.24	0.40	0.04	LRI, MS
82	65.981	1625	-	-	Unidentified 3 (Oxygenated sesquiterpene)	0.20	0.24	0.26	0.02	-
83	66.173	1627	1649	22	β-Eudesmol*	0.67	-	-	2.04	*tR*, LRI, MS
84	66.478	1631	1651	20	Pogostol	-	2.31	0.98	-	LRI, MS
85	66.515	1632	1652	20	α-Cadinol	4.53	-	-	0.80	LRI, MS
86	66.655	1634	-	-	Unidentified 4 (Oxygenated sesquiterpene)	-	0.39	-	-	-
87	67.050	1639	-	-	Unidentified 5 (Oxygenated sesquiterpene)	0.21	0.16	0.60	-	-
88	67.650	1646	-	-	Unidentified 6 (oxygenated sesquiterpene)	0.18	0.62	0.15	0.06	-
89	67.933	1650	1674	24	β-Bisabolol	-	0.81	0.05	-	LRI, MS
90	68.778	1661	1685	24	α-Bisabolol*	0.04	-	0.15	0.20	*tR*, LRI, MS
91	75.812	1777	-	-	Unidentified 7 (Oxygenated sesquiterpene)	-	-	0.36	-	-
					**Total (%)**	**92.39**	**95.32**	**95.44**	**95.11**	

* Compound identification was confirmed with reference standards. Compound identification methods: tR, identification based on the retention time (tR) of genuine compounds on the DB-5MS column; MS, identification based on mass spectra search using Wiley and NIST libraries [32,33]; LRI, identification based on the comparison of calculated linear retention indices with the literature data. LRIa: calculated retention index on DB-5MS column relative to *n*-alkanes. LRIb, retention index reported in the literature [34]. ΔLRI: difference in linear retention values (LRIb-LRIa).

Figure S2. Exploratory PCA biplot of the four essential-oil samples based on the relative peak areas of compounds present at ≥5% in at least one sample. The analysis provides a descriptive visualization of chemical similarity among the four samples and should not be interpreted as evidence of species-level chemotypes.

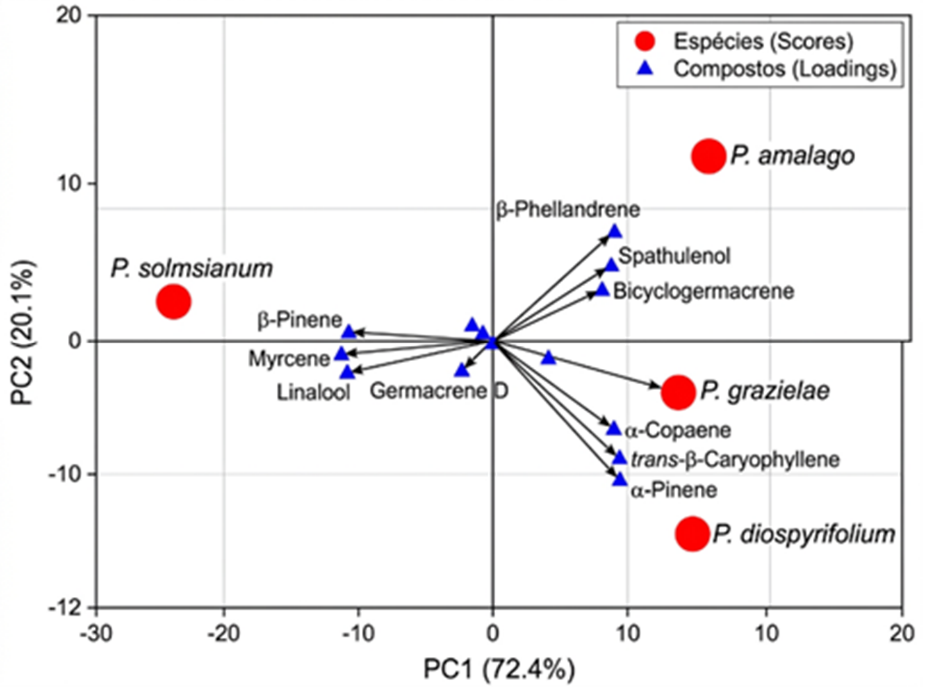

Figure S3. Exploratory hierarchical clustering dendrogram of the four essential-oil samples based on the standardized relative peak areas of compounds present at ≥5% in at least one sample. Clustering was performed using Ward's linkage and Euclidean distance. The dendrogram is intended as a descriptive representation of chemical similarity among the analyzed samples.

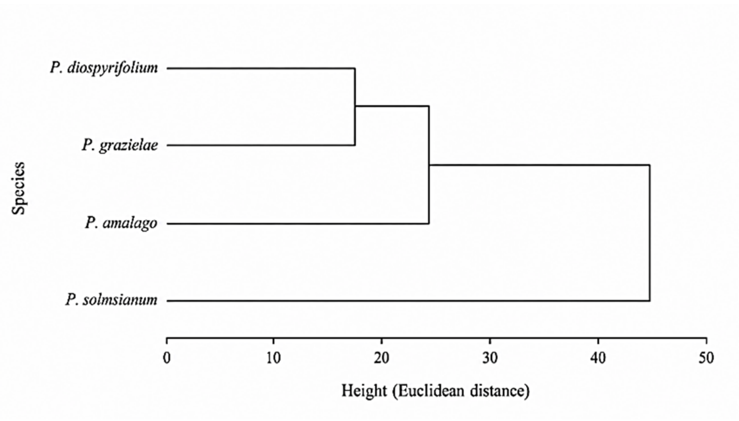

Supplement: Supplementary file 1 [file pharmaceuticals-19-01395-s001.zip › pharmaceuticals-4425470-supplementary.pdf]
